# Safety and efficacy of a new micronized formulation of the ALIAmide palmitoylglucosamine in preclinical models of inflammation and osteoarthritis pain

**DOI:** 10.1186/s13075-019-2048-y

**Published:** 2019-11-28

**Authors:** Marika Cordaro, Rosalba Siracusa, Daniela Impellizzeri, Ramona D’ Amico, Alessio Filippo Peritore, Rosalia Crupi, Enrico Gugliandolo, Roberta Fusco, Rosanna Di Paola, Carlo Schievano, Salvatore Cuzzocrea

**Affiliations:** 1 Department of Chemical, Biological, Pharmaceutical and Environmental Science, University of Messina, Messina, Italy; 2 Innovative Statistical Research srl, Prato Della Valle 24, I-35123 Padova, Italy; 3 Department of Pharmacological and Physiological Science, Saint Louis University School of Medicine, Saint Louis, USA

**Keywords:** Osteoarthritis, Pain, Mast cells, ALIAmides, N-palmitoyl-D-glucosamine, Palmitoylethanolamide, Micronization

## Abstract

**Background:**

Osteoarthritis is increasingly recognized as the result of a complex interplay between inflammation, chrondrodegeneration, and pain. Joint mast cells are considered to play a key role in orchestrating this detrimental triad. ALIAmides down-modulate mast cells and more generally hyperactive cells. Here we investigated the safety and effectiveness of the ALIAmide *N*-palmitoyl-d-glucosamine (PGA) in inflammation and osteoarthritis pain.

**Methods:**

Acute toxicity of micronized PGA (m-PGA) was assessed in rats following OECD Guideline No.425. PGA and m-PGA (30 mg/kg and 100 mg/kg) were orally administered to carrageenan (CAR)-injected rats. Dexamethasone 0.1 mg/kg was used as reference. Paw edema and thermal hyperalgesia were measured up to 6 h post-injection, when also myeloperoxidase activity and histological inflammation score were assessed. Rats subjected to intra-articular injection of sodium monoiodoacetate (MIA) were treated three times per week for 21 days with PGA or m-PGA (30 mg/kg). Mechanical allodynia and motor function were evaluated at different post-injection time points. Joint histological and radiographic damage was scored, articular mast cells were counted, and macrophages were immunohistochemically investigated. Levels of TNF-α, IL-1β, NGF, and MMP-1, MMP-3, and MMP-9 were measured in serum using commercial colorimetric ELISA kits. One- or two-way ANOVA followed by a Bonferroni post hoc test for multiple comparisons was used.

**Results:**

Acute oral toxicity of m-PGA resulted in LD50 values in excess of 2000 mg/kg. A single oral administration of PGA and m-PGA significantly reduced CAR-induced inflammatory signs (edema, inflammatory infiltrate, and hyperalgesia), and m-PGA also reduced the histological score. Micronized PGA resulted in a superior activity to PGA on MIA-induced mechanical allodynia, locomotor disability, and histologic and radiographic damage. The MIA-induced increase in mast cell count and serum level of the investigated markers was also counteracted by PGA and to a significantly greater extent by m-PGA.

**Conclusions:**

The results of the present study showed that PGA is endorsed with anti-inflammatory, pain-relieving, and joint-protective effects. Moreover, it proved that particle size reduction greatly enhances the activity of PGA, particularly on joint pain and disability. Given these results, m-PGA could be considered a valuable option in the management of osteoarthritis.

## Background

Osteoarthritis (OA) is a joint degenerative disease and a leading cause of disability in elderly populations worldwide [1], with a 104.9% rise in disability-adjusted life-years from 1990 to 2016 [2]. Once viewed as a disease primarily affecting cartilage, OA is currently considered a whole joint disease, also involving subchondral bone, synovial membrane, periarticular muscles, nerves, and ligaments [3]. According to OARSI, OA is “a disorder involving movable joints characterized by cell stress and extracellular matrix degradation initiated by micro- and macro-injury that activates maladaptive repair responses including pro-inflammatory pathways of innate immunity. The disease manifests first as a molecular derangement (abnormal joint tissue metabolism) followed by anatomic, and/or physiologic derangements (characterized by cartilage degradation, bone remodeling, osteophyte formation, joint inflammation and loss of normal joint function), that can culminate in illness” [4]. Chondrodegeneration can thus no longer be viewed as the sole pathogenetic mechanism of OA. The disease pathophysiology is much more complex and involves a vicious circle between chondrodegeneration, low-grade chronic inflammation, and pain [5]. In this triad, a special consideration has recently been paid to articular hyperactive cells, especially mast cells [6]. The latter are now believed to orchestrate neuroinflammatory processes and OA pain, suggesting they could be a new target for the treatment of OA [7–9]. ALIAmides are a class of both synthetic and naturally occurring fatty acid amides [10], whose parent molecule (i.e., *N*-palmitoyl ethanolamine, PEA) has anti-inflammatory and pain-relieving functions [11]. The main mechanism of action of ALIAmides (i.e., ALIA, autacoid local injury antagonism) mainly relies on the down-modulation of cell hyperactivity following injury [12], as first shown on mast cells by the late Nobel laureate Rita Levi Montalcini [13]. A decrease in PEA levels was reported in the synovial fluid of OA patients [14], and oral treatment with PEA has proved to benefit patients with temporomandibular joint OA and mild to moderate knee OA [15, 16]. *N*-Palmitoyl-d-glucosamine (PGA, also referred to as Glupamid) is one of the less studied among ALIAmides, and only few reports on this compound have been published so far [17–19]. Chemically speaking, PGA is the amide of palmitic acid and glucosamine and a highly lipophilic compound with a predicted logP value of 5.6 [20]. Similarly to fatty acid amides, it is hydrolysed by fatty acid amide hydrolases [21, 22], resulting in the intracellular release of glucosamine. PGA might thus exert a dual effect, i.e., the ALIA effect down-modulating cell hyperactivity [18] and the putative effect of glucosamine on the anabolic/catabolic balance of cartilage [23, 24]. These actions well fit within the new understanding of OA pathogenesis, and PGA may help overcome currently unmet needs in the treatment of OA. The aim of this study was to evaluate the anti-inflammatory, chondroprotective, and pain-relieving effects of PGA and investigate whether micronization could improve the effects of this highly lipophilic compound, similarly to findings obtained with the congener PEA [25, 26]. An acute toxicity study on micronized PGA (m-PGA) was performed prior to the main study, in order to investigate its safety.

## Methods

### Animals

This study was performed on Sprague-Dawley male (200–230 g, 7 weeks old) and female (230–250 g, 8 weeks old) rats supplied by Envigo RMS Srl, S. Pietro al Natisone, Udine, Italy. Nulliparous and non-pregnant female rats were used for toxicity study, since females are generally slightly more sensitive than males to toxic effects. Rats were housed in individual polycarbonate cages (two for each group) and maintained under a 12:12 light-dark cycle at 21 ± 1 °C and 50 ± 5% humidity. The cage bedding material was corn cob and was changed twice per week. Food and water were available ad libitum. The study was approved by the University of Messina Review Board for the care of animals. Animal care was in accordance with Italian regulations on protection of animals used for experimental and other scientific purposes (D.M.116192) as well as with EEC regulations (O.J. of E.C. L 358/1 12/18/1986) and in compliance with the requirements of Italian Legislative Decree no. 26/2014 and subsequent guidelines issued by the Italian Ministry of Health on March 16, 2015.

### Reagents

PGA and micronized PGA (m-PGA, particle size from 0.6 to 10 μm) were kindly provided by Epitech group SpA (Saccolongo, Italy). All other compounds were obtained from Sigma-Aldrich, Milan, Italy. All chemicals were of the highest commercial grade available. All stock solutions were prepared in non-pyrogenic saline (0.9% NaCl, Baxter International, Rome, Italy).

### Experimental design

The experiment was divided in two steps. First, acute toxicity testing was conducted. Then, the effect of micronization on the activity of PGA was evaluated in two standard models of inflammation and OA pain, subplantar injection of carrageenan (CAR), and intra-articular injection of sodium monoiodoacetate (MIA) respectively.

### Acute toxicity testing

To date, no experimental data have been published concerning PGA safety. In the present study, we thus assessed the acute toxicity of m-PGA administered by gavage using a stomach tube as per OECD Guideline No.425 [27]. This method permits estimation of an LD50 and minimizes the numbers of animals used. In particular, the limit test, i.e., a sequential test that uses few animals, is suggested for studying compounds likely to be nontoxic, as it is the case here. Actually, micronized PEA has LD50 > 2000 mg/kg [28], and LD50 of oral glucosamine is approximately 8000 mg/kg with no adverse effects at 2700 mg/kg for 12 months [29]. The limit test was used accordingly for m-PGA. Under this procedure, one animal is dosed at the limit dose (i.e., 2000 mg/kg), and if it survives, additional animals are dosed sequentially, at 48-h intervals. Animals are observed with a special attention given during the first 4 h and daily thereafter, for a total of 14 days [27]. Results to be recorded include body weight, observations of any sign(s) of toxicity, and any gross pathological changes. The LD50 is greater than 2000 mg/kg if three or more animals survive after 14 days [27]. The complete guideline is available at https://www.oecd-ilibrary.org/content/publication/9789264071049-en).

Rats were divided into the following experimental groups:

- Control group (a single dose by oral gavage of carboxymethylcellulose 1%, *n* = 6).
- m-PGA group (a single dose by oral gavage 2000 mg/kg body weight, of m-PGA dissolved in an aqueous suspension of carboxymethylcellulose 1%, n = 6).

Body weight, mortality, signs of toxicity (i.e., tremors, convulsions, salivation, diarrhea, lethargy, sleep, and coma) and gross pathological changes of all animals were recorded and evaluated. At the end of the study (i.e., 14 days after administration of the tested compound/vehicle), the animals were sacrificed and the samples were collected. Briefly, paraffin tissue sections (thickness, 7 μm) were deparaffinized with xylene, stained with hematoxylin and eosin, and studied by light microscopy (AxioVision, Zeiss, Milan, Italy) by an experienced histopathologist. Each histological analysis was completed in a blinded fashion. Tissue sections of brain, spinal cord, heart, liver, kidney, bladder, lung, uterus, stomach, colon, and bowel were examined.

### Experimental models

#### CAR-induced inflammation

Rats were anesthetized with 5.0% isoflurane in 100% O_2_ (Baxter International, Rome, Italy) and received a subplantar injection of CAR (0.1 ml/rat of a 1% suspension in saline) (Sigma-Aldrich, Milan, Italy) with a 27-gauge needle into the right hind paw, as previously described [30, 31]. The animals were sacrificed at 6 h post CAR injection by isoflurane overdose. All analyses conducted in CAR-injected rats were performed in a blinded manner.

#### MIA induction model

OA was induced by intra-articular injection of MIA in the right knee joint as previously described [31, 32]. Briefly, rats were anesthetized with 5.0% isoflurane in 100% O_2_ and a volume of 25 μl saline + 3 mg of MIA was injected into the knee joint. The left knee received an equal volume of saline. MIA was prepared in sterile conditions and injected into the joint using a 50 μl Hamilton syringe with a 27-gauge needle. On day 21 post-MIA injection, rats were sacrificed by anesthetic overdose and perfused with 4% paraformaldehyde. All analyses conducted in MIA-injected rats were performed in a blinded manner.

### Treatment

To study whether micronization affected the activity of PGA, rats were divided into the following treatment groups, each compound being administered orally by gavage (i) as a single administration 30 min before CAR injection (*N* = 6/group) or (ii) as a repeated administration three times per week for 21 days, starting the third day after MIA injection (*N* = 10/group). Before administration, PGA—whether micronized or not—was dissolved in carboxymethylcellulose (1% w/v in saline). In CAR-induced inflammation, dexamethasone (in the sodium phosphate salt form) was used as reference and dissolved in saline. Saline was used as vehicle. Treatment groups were as follows.

1. CAR + vehicle: rats were subjected to CAR-induced paw edema, as described above and administered vehicle;
2. CAR *+* PGA 30: same as the CAR + vehicle group but non-micronized PGA 30 mg/kg was administered instead of vehicle;
3. CAR *+* PGA 100: same as the CAR + vehicle group but non-micronized PGA 100 mg/kg was administered instead of vehicle;
4. CAR *+* m-PGA 30: same as the CAR + vehicle group but micronized PGA 30 mg/kg was administered instead of vehicle;
5. CAR *+* m-PGA 100: same as the CAR + vehicle group but micronized PGA 100 mg/kg was administered instead of vehicle;
6. CAR *+* Dex: same as the CAR + vehicle group but dexamethasone 0.1 mg/kg was administered instead of vehicle;
7. Control group: saline was administered instead of CAR.
8. MIA + vehicle: rats were subjected to MIA intra-articular injection, as described above and administered vehicle;
9. MIA *+* PGA 30: same as the MIA + vehicle group but non-micronized PGA 30 mg/kg was administered instead of vehicle;
10. MIA *+* m-PGA 30: same as the MIA + vehicle group but micronized PGA 30 mg/kg was administered instead of vehicle;
11. Control group: saline was administered instead of MIA.

Doses were chosen based on a dose-response study carried out in our lab. PGA at 30 mg/kg dose only was used in MIA-induced OA pain, given that the higher dose (whether micronized or not) did not show any advantage over the lower one in CAR-induced inflammation.

### Assessment of CAR-induced paw edema

Edema was expressed as increase in paw volume (ml) after CAR injection relative to pre-injection value and measured using a plethysmometer (Ugo Basile, Varese, Italy), as previously described [30, 31]. Paw volume was measured immediately prior to CAR injection and thereafter at 30 min and hourly intervals for 6 h.

### Pain-related behavioral analysis in the CAR-induced inflammation

The hyperalgesic response to heat was determined at different time points (0, 30 min, and hourly intervals for 6 h) based on the method described by Hargreaves et al. [33], using a Basile Plantar Test (Ugo Basile, plantar test apparatus 7371; power requirement: 230-115 V, 60-50 Hz, 60VA maximum) as previously described [31]. Results are expressed as paw withdrawal latency changes (seconds).

### Histological analysis following CAR injection

Histological analysis of hematoxylin and eosin-stained paw tissue, collected 6 h after intraplantar CAR injection, was performed as previously described [31]. Briefly, the degree of tissue damage was evaluated according to Bang and Coll [34]., on a 6-point score, from 0 (no inflammation) to 5 (severe inflammation). The photographs obtained (*n* = 5 photos from five slides for each sample) were collected from all animals in each experimental group. The histological coloration (five slides for each same sample) was repeated three times on different days.

### Myeloperoxidase (MPO) activity following CAR injection

MPO activity, an index of neutrophilic granulocyte infiltration, was evaluated as previously described [31]. Briefly, the rate of change in absorbance was measured spectrophotometrically at 650 nm. MPO activity was measured as the quantity of enzyme degrading 1 mM of peroxide min^− 1^ at 37 °C and expressed in units per gram of wet tissue weight.

### Assessment of MIA-induced mechanical allodynia

Mechanical allodynia was evaluated using a dynamic plantar Von Frey hair esthesiometer on day 0 and 3, 7, 14, and 21 days post-injection (Ugo Basile, Comerio, Italy) as previously described [31]. Briefly, when the esthesiometer was activated, the Von Frey–type 0.5-mm filament began to move below the metatarsal region, with a gradually increasing force on the plantar surface, starting below the threshold of detection and increasing until the stimulus became painful and the rat removed its paw. The force required to produce a paw withdrawal reflex (i.e., the paw withdrawal threshold, PWT) and the time interval between the stimulus and the response (i.e., paw withdrawal latency, PWL) were automatically detected and recorded (in grams and seconds respectively). A maximum force of 50 g and a ramp speed of 20 s were used for all the esthesiometry tests.

### Motor function analysis following MIA injection

Motor functional recovery of the rear limb was evaluated by walking track analysis, a reliable and easily quantifiable noninvasive method based on gait analysis by means of specific footprint parameters as previously described [31]. Walking track analysis was performed before MIA injection and 3, 7, 14, and 21 days post-injection. From the footprints, several measurements are taken between different anatomic landmarks and then incorporated in a mathematical formula. More specifically, the measurements were the following: (i) the print length (PL, i.e., the distance from the heel to toe), (ii) the toe spread (TS, i.e., the distance from the first to the fifth toes), (iii) the intermediary toe spread (IT, i.e., the distance from the second to fourth toes). These measures were recorded for the MIA-injected and contralateral normal limb with the prefix E and N being added respectively. The motor functional recovery was calculated using the following formula, whose numerical value is termed SFI (sciatic functional index): − 38.3 [(EPL − NPL)/NPL] + 109.5 [(ETS-NTS)/NTS] + 13.3 [(EIT − NIT)/NIT] − 8.8 [35]. SFI values in the control group were assumed as zero.

### Histological analysis of MIA-injected rats

The MIA- and vehicle-injected tibiofemoral joints were dissected immediately after sacrifice and post-fixed in neutral buffered formalin (containing 4% formaldehyde) as previously described [31]. Mid-coronal tissue sections (5 μm) were stained with H/E, observed using a Leica DM6 microscope at × 10 magnification (Leica Microsystems SpA, Milan, Italy) equipped with a motorized stage and associated with Leica LAS X Navigator software (Leica Microsystems SpA, Milan, Italy). Collagen content was assayed according to the manufacturer’s protocol (Bio-Optica, Italy, Milan), with tissue sections being stained with Masson’s trichrome. Proteoglycan depletion and cartilage destruction were assayed using Safranin O/Fast green staining. The number and degranulation extent of mast cells were also assessed in tissue sections stained with toluidine blue as previously described [36].

Finally, a modified Mankin histologic scoring system was used to evaluate cartilage damage, from 0 (normal histology) to 12 (complete disorganization and hypocellularity) [37]. The photographs obtained (*n* = 5 photos from five slides for each sample) were collected from all animals in each experimental group. The histological coloration (five slides for each same sample) was repeated three times on different days.

### Radiographic analysis of MIA-injected rats

Radiographic analysis was performed by X-ray (Bruker FX Pro instrument, Milan, Italy). Radiographs were evaluated by an expert observer and scored using a semi-quantitative grading scale as previously indicated [38]. Briefly, the study features were scored as follows: joint space, from 0 (normal) to 3 (complete loss of joint space); subchondral bone sclerosis, from 0 (normal) to 3 (severe); osteophyte formation, from 0 (normal) to 3 (osteophytes present on both tibial and femoral condyle).

### Serum concentration of inflammatory, nociceptive, and matrix degradation markers following MIA injection

On day 21 post-MIA injection, rats were sacrificed and serum were taken and stored at − 80 °C. Subsequently, the concentration of tumor necrosis factor alpha (TNF-α), interleukin-1beta (IL-1β), nerve growth factor (NGF), and matrix metalloproteinase-1-3-9 (MMP-1, MMP-3, MMP-9) were measured in serum using commercial colorimetric ELISA kits (TNF-α, IL-1β, and NGF: Thermo Fisher Scientific, DBA s.r.l. Milan Italy; MMP-1, MMP-3, and MMP-9: Cusabio, DBA s.r.l. Milan Italy).

### Immunohistochemistry in CAR- and MIA-injected rats

Paw and joint knee sections were immunostained with CD68 as previously described by Cordaro and coll [39].. Slices were incubated overnight with anti-CD68 antibody (1:100 in PBS, v/v, Santa Cruz Biotechnology, Heidelberg, Germany). All immunohistochemical analyses were carried out by an observer blind to treatments.

### Data analysis

Data are expressed as mean ± standard error of the mean (SEM) of *N* observations, *N* representing the number of animals analyzed, with the exception of the ordinal level variable (i.e., histological score), for which median and range were used. In experiments involving histology, the figures are representative of at least three independent experiments performed on different days. The results were analyzed by one- or two-way ANOVA followed by a Bonferroni post hoc test for multiple comparisons. Kruskal-Wallis test followed by Dunn’s test for post hoc comparisons with Bonferroni-Holm *p* correction was used for the histological score, due the ordinal level nature of the variable (i.e., 0- to 5-point scale). Data were analyzed using SAS v9.2 (SAS Institute, Cary, NC, USA). The significance threshold was set at 0.05. Exact *p* values are reported, unless less than 1 out of 10,000 (reported as *p* < 0.0001), 0.0001 being the lower limit for the statistical program.

## Results

### Oral toxicity

**Fig. 1:**
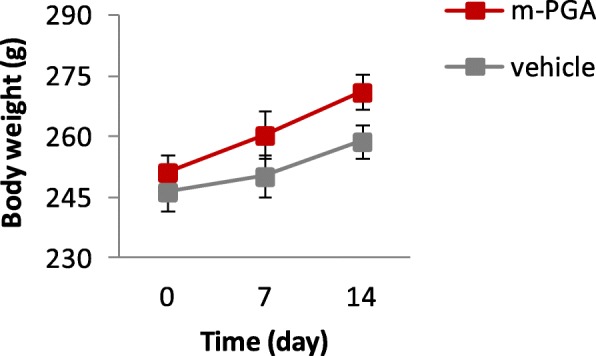
Acute oral toxicity test. Body weight was recorded weekly. No difference was observed in body weight change between m-PGA- and vehicle-treated groups

A single oral dose of m-PGA at an upper dose of 2000 mg/kg did not cause any mortality or clinical signs in the acute oral toxicity test in female rats. In addition, no difference in body weight was registered between groups (Fig. 1). Finally, no important histological alterations compared to the vehicle-treated group were observed (Additional file 1: Figure S1 and Additional file 2: Figure S2). Acute toxicity by oral route resulted to a LD50 cut-off value of 2000 mg/kg b.w.

### Effect of m-PGA on CAR-induced inflammation

**Fig. 2:**
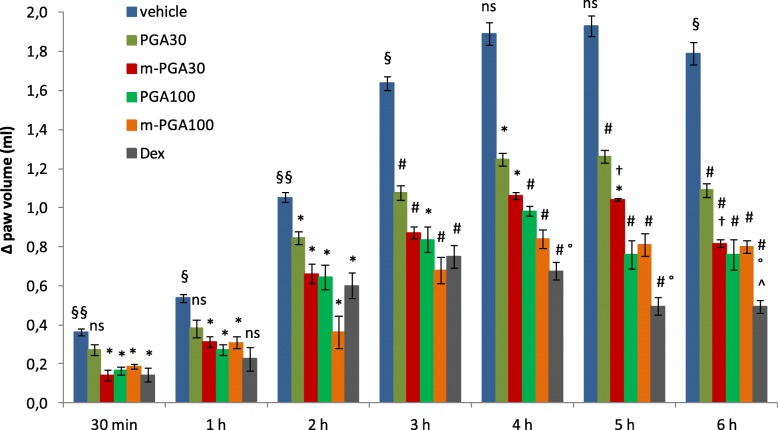
Anti-inflammatory effect of m-PGA. Inflammation was assessed as increase in paw volume (ml) after CAR injection relative to pre-injection value. Results are expressed as means ± SEM (*N* = 6/group). *p < 0.05 and #*p* < 0.0001 vs vehicle; §*p* < 0.05 and §§*p* < 0.0001 vs previous time point (analysis performed only on vehicle-treated group); †*p* < 0.05 vs non-micronized PGA (equal dose); °*p* < 0.05 vs m-PGA30; ^*p* < 0.05 vs m-PGA100; ns: not significant

**Fig. 3:**
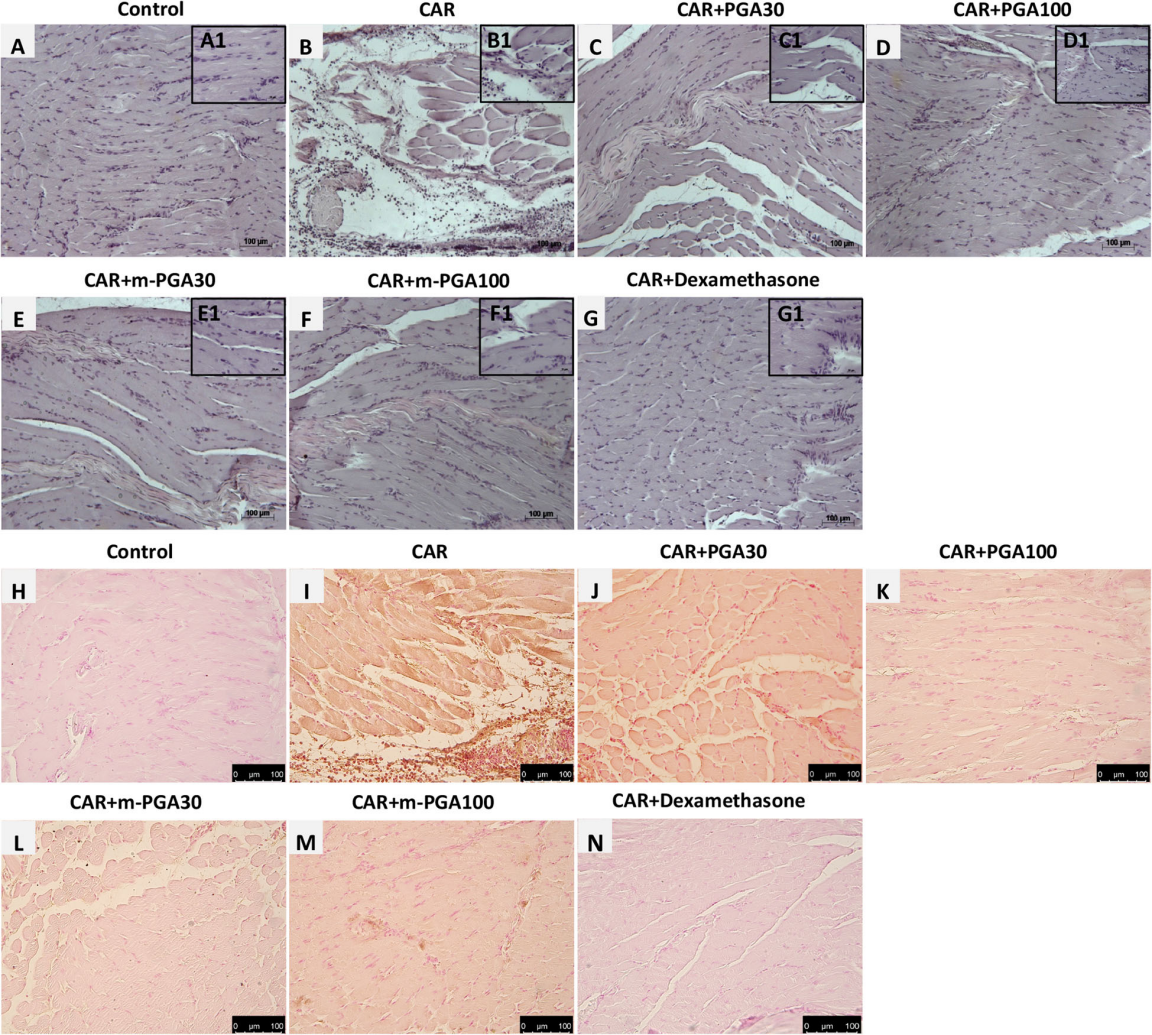
Effect of m-PGA on CAR-induced inflammatory changes. The effect of the study treatments was assessed histologically in H/E stained sections (**a**–**g**) and using immunohistochemical staining of CD68 (**h**-**n**). Control animals exhibiting a normal architecture of the paw (**a**). On the contrary, paw biopsies after CAR injection showed a marked edema, with an important tissue alteration and with a pronounced inflammatory cellular infiltration (**b**). Treatment with PGA (**c**, **d**), and better with m-PGA (**e**, **f**), significantly reduced the pathological changes in the tissues. The same beneficial effect was produced by dexamethasone treatment (**g**). CD68 immunostaining showed that the activation of macrophages CAR-induced (**i**) was significantly decreased after PGA treatment (**j**, **k**), even more so by m-PGA (**l**, **m**) and dexamethasone (**n**). No positive staining was founded in control animals (**h**). See Methods for further details

The anti-inflammatory potential of the tested compounds was estimated through inhibitory effect on (i) CAR-induced paw, (ii) MPO enzymatic activity, and (iii) histological degree of tissue inflammation. Intraplantar injection of CAR led to a significant time-dependent increase in paw volume (*p* < 0.0001), that was significantly limited by each treatment (see Fig. 2 for significance levels). At the latest time points, m-PGA (at 30 mg/kg but not 100 mg/kg) showed a significantly superior effect compared to non-micronized PGA (equal dose, *p* < 0.05). Finally, dexamethasone only occasionally outweighed the anti-inflammatory effect of m-PGA (both doses, Fig. 2). Neutrophil infiltration as measured by MPO activity significantly increased 6 h after CAR injection (*p* < 0.0001), the increase being highly and significantly counteracted by each of the tested compounds (Table 1; *p* < 0.0001 for all comparisons) with dexamethasone showing statistically significant superiority over all the other treatments except for PGA100. Data and comparisons are detailed in Table 1. CAR paw injection led to a marked infiltration of inflammatory cells (Fig. 3). In particular, a greater amount of CD68-positive macrophages compared to control was observed (Fig. 3h, i). The increase was counteracted by PGA at both doses (Fig. 3j, k) and even more so by m-PGA and dexamethasone (Fig. 3l–m). Only m-PGA (both doses) and dexamethasone significantly reduced the histological score, as detailed in Table 2.

**Table 1:** Effect of the study treatments on CAR-induced neutrophil infiltration

	Control	Vehicle	PGA30	m-PGA30	PGA100	m-PGA100	Dex
*N*	6	6	6	6	6	6	6
Min	12.4	2028.2	615.5	406.9	235.5	379.3	151.9
Max	38.1	2598.3	991.5	588.2	525.8	517.3	291.2
*Mean*	*27.3*^*#*^	*2343.9*	*773.7*^*#*^	*491.6*^*#*^	*381.3*^#*^	*438.5*^#*^	*223.2*^#*^^
SEM	34	81.5	69.6	31.5	42.8	20.6	22.4

Neutrophil infiltration was evaluated through MPO activity in the different treatment groups at the sixth hour post CAR injection. Values are expressed as units of MPO per gram of wet tissue. #*p* < 0.0001 vs Vehicle, *p < 0.05 vs PGA30, ^*p* < 0.05 vs m-PGA (either doses)

**Table 2:** Effect of the study treatments on CAR-induced histological damage

	Control	Vehicle	PGA30	m-PGA30	PGA100	m-PGA100	DEX
*N*	6	6	6	6	6	6	6
Min	0	3	2	1	1	1	0
*Median*	*0*^*#*^	*4.5*	*2.5*	*1*^*§*^	*2*	*1*^*§*^	*1*^*§*^
Max	0	5	3	2	2	2	2
StdErr	0	0.3	0.2	0.2	0.2	0.2	0.3
*P* (vs CAR)	< 0.0001	–	1.0000	0.0442	0.2244	0.0442	0.0030

The histologic severity score was assessed according to Bang and Coll [34]., on a 6-point scale from 0 (no inflammation) to 5 (severe inflammation). #*p* < 0.0001 vs Vehicle, §*p* < 0.05 vs Vehicle

### Effect of m-PGA on CAR-induced inflammatory pain

**Fig. 4:**
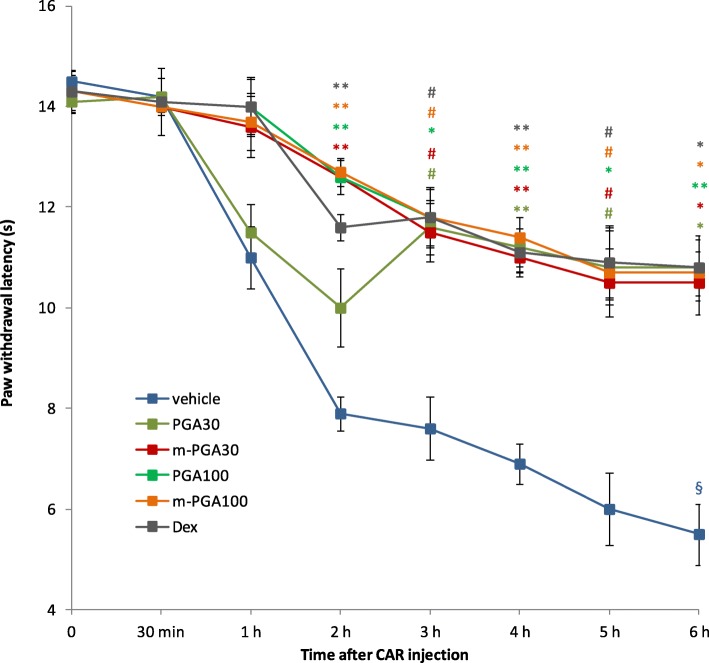
Effect of m-PGA on inflammatory pain. Pain was assessed by determining CAR-induced thermal hyperalgesia, as measured by paw withdrawal latency. Results are expressed as means ± SEM (*N* = 6/group) of paw withdrawal latency changes (seconds). See Methods for further details. §*p* < 0.0001 vs baseline (analysis performed only on vehicle-treated group); #*p* < 0.05, *p < 0.01, and **p < 0.001 vs vehicle (colors correspond to those specified in the legend). No symbol: not significant

Intraplantar injection of CAR led to a time-dependent hyperalgesia, as shown by the statistically significant decrease in paw withdrawal latency in the vehicle-treated group (*p* < 0.0001, Fig. 4). Oral treatment with PGA, m-PGA, and dexamethasone significantly counteracted CAR-induced hyperalgesia starting from the second (third for PGA at 30 mg/kg) and up to the sixth hour post CAR, as evidenced by the significantly increased latency times compared to the vehicle-treated group (Fig. 4). No significant difference between treatment groups was observed at any time point, PGA being equally active compared to dexamethasone regardless of the dose and particle size.

### Effect of m-PGA on pain and motor function deficits following MIA induction

**Fig. 5:**
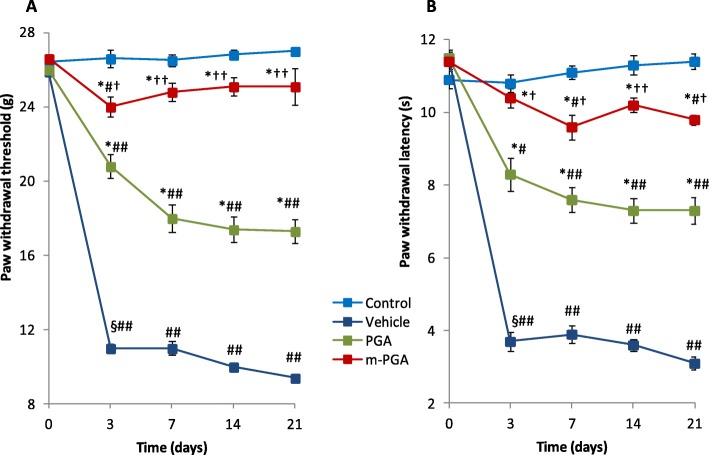
Pain-relieving effect of m-PGA on MIA-induced mechanical allodynia. Results are expressed as means ± SEM (*N* = 10/group) of paw withdrawal threshold (**a**) and paw withdrawal latency (**b**). See Methods for further details. §*p* < 0.0001 vs previous time point (analysis performed only on vehicle-treated group); *p < 0.0001 vs vehicle; #*p* < 0.05 and ##*p* < 0.0001 vs control (i.e., saline-injected animals); †*p* < 0.05 and ††*p* < 0.0001 vs non-micronized PGA

**Fig. 6:**
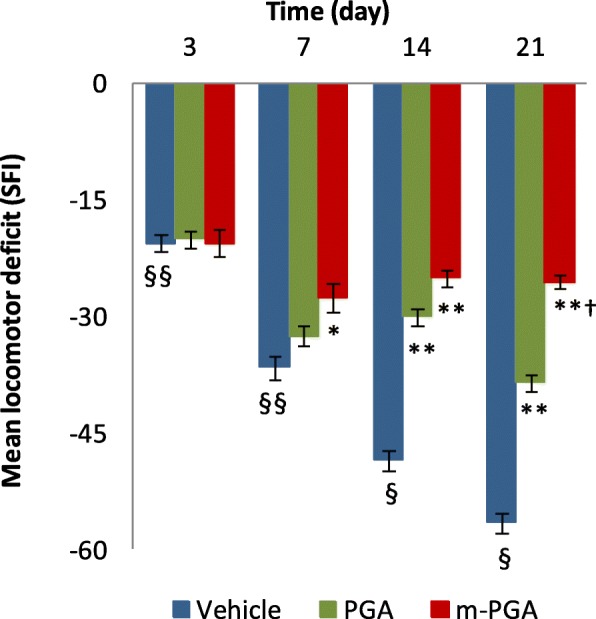
Effect of m-PGA on MIA-induced locomotor deficit. The locomotor function at 3, 7, 14, and 21 days after MIA injection was assessed through walking track analysis and expressed as functionality index of the sciatic nerve (SFI). Values close to 0 indicate normal function while values tending to − 100 indicate total impairment. Results are expressed as means ± SEM (*N* = 10/group). §*p* < 0.05 and §§*p* < 0.0001 vs previous time point; *p < 0.05 and **p < 0.0001 vs vehicle; †*p* < 0.0001 vs non-micronized PGA

Because pain is the hallmark of MIA-induced OA, mechanical allodynia in MIA-injected rats was assessed. In the von Frey hair assessment test, PWT and PWL decreased in the vehicle-treated group compared to controls (*p* < 0.0001 for both variables at every time point, Fig. 5a, b). The decrease in PWT ranged from 58% (day 3) to 65% (day 21). PGA and m-PGA significantly counteracted MIA-induced allodynia (*p* < 0.0001at every time point and all comparisons), limiting the reduction in PWT to values not exceeding 36% (PGA) and 10% (m-PGA). Notably, PWT values in the m-PGA-treated group did not differ from those observed in the control group at any time point, suggesting that the micronized compound completed reversed the effect of MIA injection on PWT. Similar results were observed on PWL, latencies being reduced by 64% (day 3) to 73% (day 21) in the vehicle-treated group, while percentage reduction did not exceed 14% in the m-PGA-treated group. Treatment with m-PGA resulted in a superior activity to PGA on both PWT (*p* = 0.0273, *p* < 0.0001, *p* < 0.0001, and *p* = 0.0001) and PWL (*p* = 0.0196, *p* = 0.0106, *p* = 0.0001, and *p* = 0.0009) at days 3, 7, 14, and 21 respectively (Fig. 5a, b). In addition, motor function at different time points was assessed by walking track analysis. In the vehicle-treated group, SFI values were significantly lower than zero (i.e., control group), locomotor function being increasingly impaired at days 3, 7, 14, and 21 (Fig. 6) (*p* < 0.0001 at all time points). Treatment with PGA improved locomotor function starting at day 14 (*p* < 0.0001), while a statistically significant earlier effect was observed in the m-PGA-treated group already after 7 days (*p* = 0.0274). At the study end, the improvement in locomotor function was significantly higher in the m-PGA- compared to PGA-treated group (*p* < 0.0001, Fig. 6).

### Effect of m-PGA on histologic and radiographic MIA-induced joint damage

**Fig. 7:**
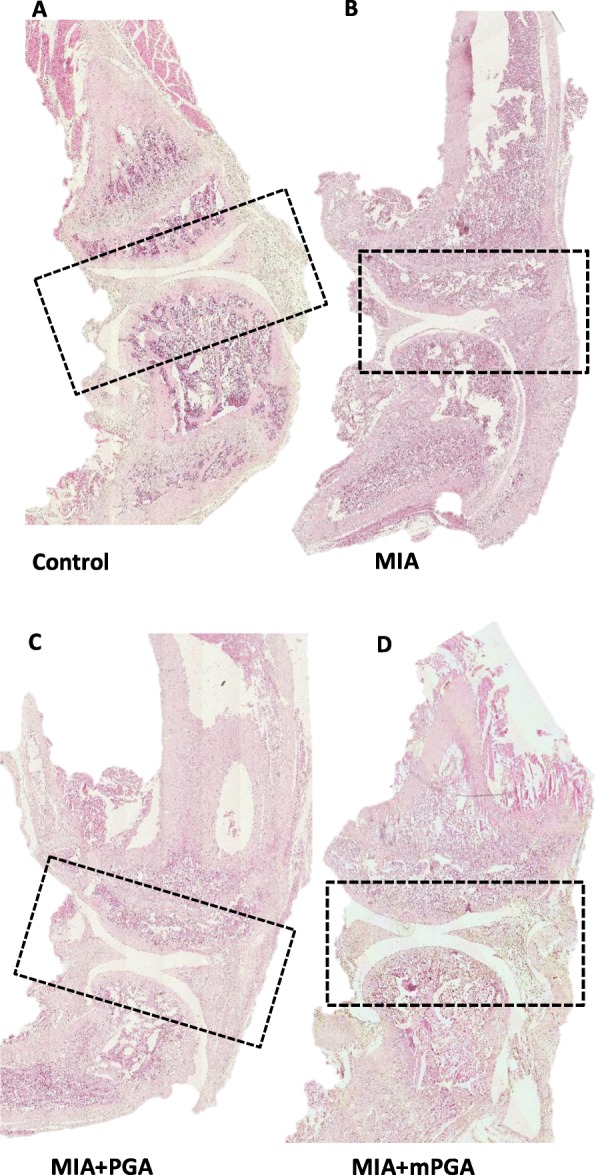
Protective effect of m-PGA- on MIA-induced histologic damage. Representative images of H/E-stained sections. Rat knee joints injected with MIA showed a significant expansion of the synovial membrane probably caused by proteinaceous edema fluid and fibrin with infiltrating macrophages, plasma cells, lymphocytes, and neutrophils (**b**) compared to control (**a**). Following PGA (**c**) and even more so by m-PGA (**d**), synovial membrane expansion and cellular infiltrate still present to a lesser degree. Dotted boxes indicate the region of interest

**Fig. 8:**
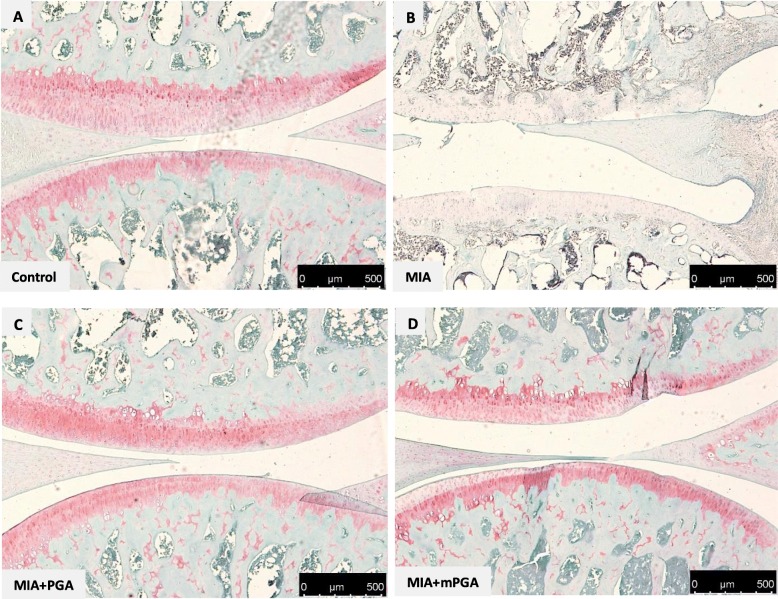
Effect of m-PGA on MIA-induced cartilage degeneration. Safranin O-fast green staining of articular cartilage in the knee joint of MIA-injected animals (**b**) showed a decrease in the proteoglycan content (red staining) as well as erosion and roughening of the articular cartilage and loss of the superficial zone compared to controls (**a**). PGA (**c**) and even more so m-PGA (**d**) treatment was able restore the histological appearance of the native cartilage (i.e., restored red staining)

**Fig. 9:**
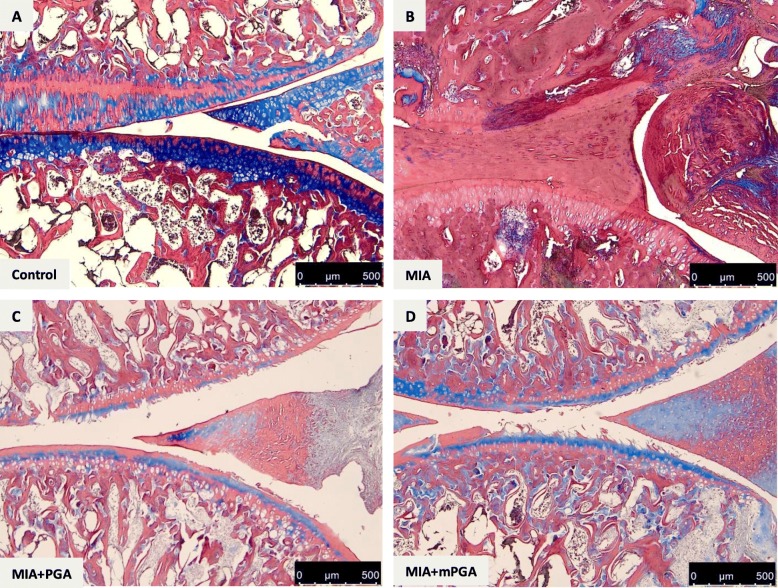
Effect of m-PGA on MIA-induced collagen changes (Masson trichrome stain). After MIA injection, the knee joint presented cartilage surface fibrillation and thinning, with decreased collagen content (blue staining) and cleft formation, as well as fattened subchondral plate, massive subchondral sclerosis, and a more disoriented trabecular bone structure (**b**) compared to control (**a**). PGA (**c**) and m-PGA (**d**) counteracted these changes, as also evidenced by the increased and more organized blue staining

**Fig. 10:**
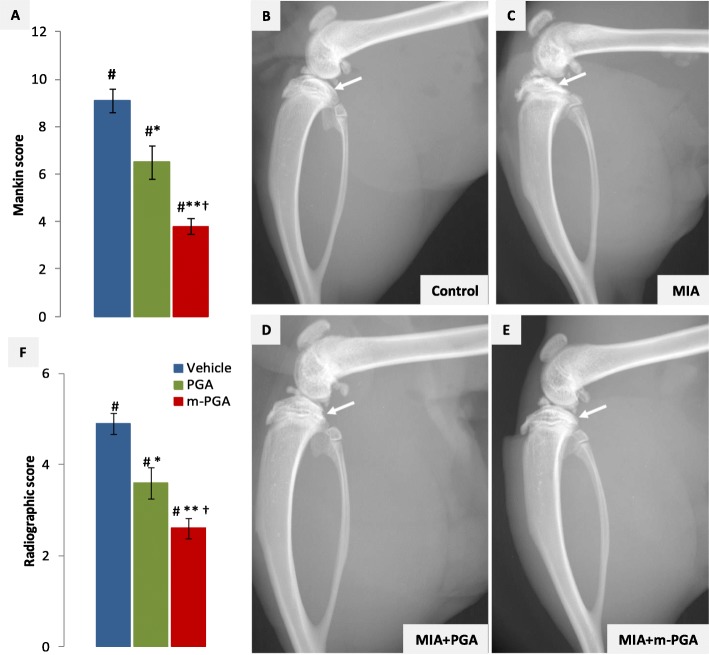
Protective effect of m-PGA- on MIA-induced joint damage. In order to score the severity of the osteochondral damage of the tibiofemoral joint, a modified Mankin score from 0 (normal histology) to 12 (complete disorganization and hypocellularity) (**a**) and a semi-quantitative radiographic scale from 0 (normal) to 9 (osteoarthritis) (**f**) were used (see Methods for further details). To evaluate the bone changes of knee joints after MIA injection, radiographic examination was performed on OA rat treated with PGA, m-PGA, and vehicle. As shown in Fig. 10b, the surface of knee joints was regular in the control group. In contrast, loss of joint space, incomplete and thickening articular surface with sclerosis and deformation were observed in MIA-injected rats (**c**). The **d** PGA- and **e** m-PGA-treated groups which markedly prevented these alterations at the knee joint were observed. Results are expressed as means ± SEM (*N* = 10/group). Animals in the control group were scored 0 at either scales (data not shown). *p < 0.05 and **p < 0.0001 vs vehicle; #*p* < 0.0001 vs control; †*p* < 0.0001 vs non-micronized PGA. Results are expressed as means ± SEM (*N* = 10/group)

Histological examination of hematoxylin/eosin-, Masson’s trichrome-, and Safranin O/Fast green-stained knee sections 21 days after intra-articular injection of MIA revealed an increase in irregularities in the surface layer, and multi-layering in transition and radial zones of the cartilage (Fig. 7 a, b), as well as collagen and proteoglycan loss compared to controls (Figs. 8 and 9a, b). The Mankin score deteriorated accordingly (9.1 ± 0.5, *p* < 0.0001; Fig. 10a). Similarly, radiographic analysis showed severe joint damage around tibia and femur in MIA group with articular cartilage erosion, loss of joint space, subchondral bone sclerosis (Fig. 10b, c) and a significantly higher radiographic score compared to controls (4.9 ± 0.23 vs 0, *p* < 0.0001; Fig. 10f). The extent of cartilage abnormalities, collagen degradation, and proteoglycan loss was reduced by PGA (panel c, Figs. 7, 8, and 9) and even more so by m-PGA (panel d, Figs. 7, 8, and 9), as reflected by the significant reduction of the histological severity score following PGA (6.5 ± 0.70, *p* = 0.0083) and m-PGA (3.8 ± 0.33 *p* < 0.0001) treatment (Fig. 10a). The radiographic severity score decreased accordingly following PGA (3.6 ± 0.34, *p* = 0.0124) and m-PGA (2.6 ± 0.22, *p* < 0.0001; Fig. 10f). Notably, the micronized formulation displayed superior activity over the naïve one (*p* = 0.0084 and *p* = 0.0258 for the comparisons of the effects on the histologic and radiographic score respectively; Fig. 10a, f).

### Effects of m-PGA on plasma concentration of inflammatory, nociceptive and matrix degradation markers

MIA injection significantly elevated serum levels of all the investigated markers (*p* < 0.0001, Table 3). PGA treatment exerted a small but significant inhibition of MIA-induced increase (from 6 to 22% inhibition, *p* < 0.05 for all the investigated cytokines and growth factors), while m-PGA displayed a significantly greater effect (*p* < 0.0001), with an inhibitory power ranging from 37 to 62% depending on the marker (Fig. 11).

**Table 3:** Effect of MIA injection on plasma concentration of inflammatory, nociceptive, and matrix degradation markers in vehicle-treated animals

		IL-1	MMP-1	MMP-3	MMP-9	NGF	TNF-α
Control	*N*	10	10	10	10	10	10
	Min	0.03	18	11	11	178	0.1
	Max	0.06	26	20	21	191	0.5
	Mean	*0.04*	*22.7*	*15.6*	*15.9*	*185.3*	*0.30*
	SEM	0.00	1.08	1.06	1.19	1.65	0.04
Vehicle	*N*	10	10	10	10	10	10
	Min	0.14	32	73	62	326	0.9
	Max	0.18	47	92	77	388	1.4
	Mean	*0.16*^*#*^	*40.5*^*#*^	*83.7*^*#*^	*69.7*^*#*^	*363.3*^*#*^	*1.06*^*#*^
	SEM	0.01	1.64	1.84	1.37	5.44	0.05

#*p* < 0.0001 vs Control

**Fig. 11:**
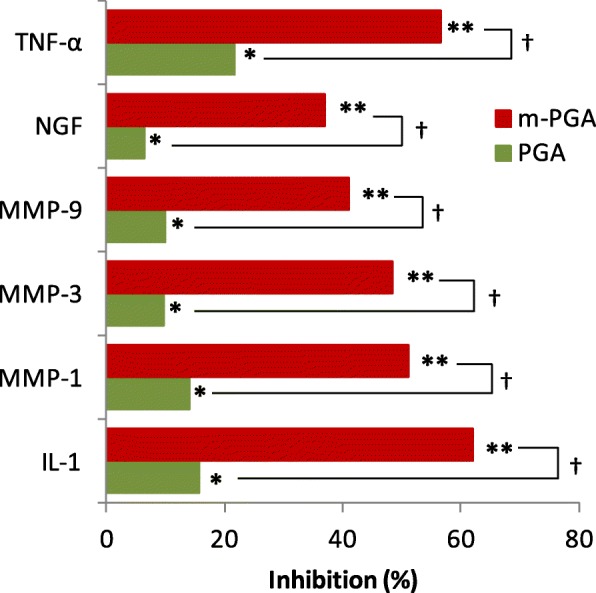
The inhibitory effect of m-PGA on MIA-induced increase in the serum levels of cytokines, metalloproteinases, and NGF. Mean percentage inhibition was calculated as follows: [(Lc − Lt)/Lc] × 100, where Lc = mean serum level in the vehicle group and Lt = mean serum level in the treated group. *p < 0.05 vs vehicle; **p < 0.0001 vs vehicle; †*p* < 0.0001 comparison between PGA and m-PGA

### Effect of m-PGA on joint inflammatory cell hyper-activation following MIA injection

**Fig. 12:**
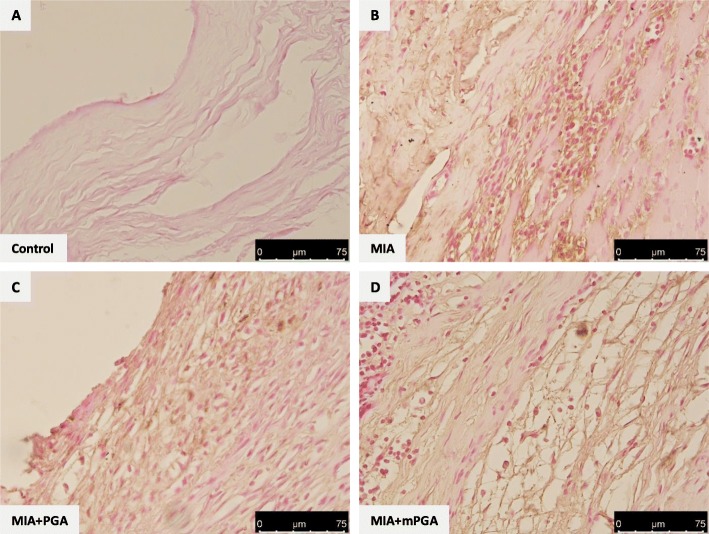
Effect of m-PGA on MIA-induced macrophage accumulation. Synovial tissue from the MIA group (**b**) showed markedly positive staining for CD68+ antibody compared the control group (**a**). PGA (**c**) and even more so m-PGA group (**d**) showed weaker immunoreactivity, suggesting that both treatments reduced accumulation of macrophages in the joint

**Fig. 13:**
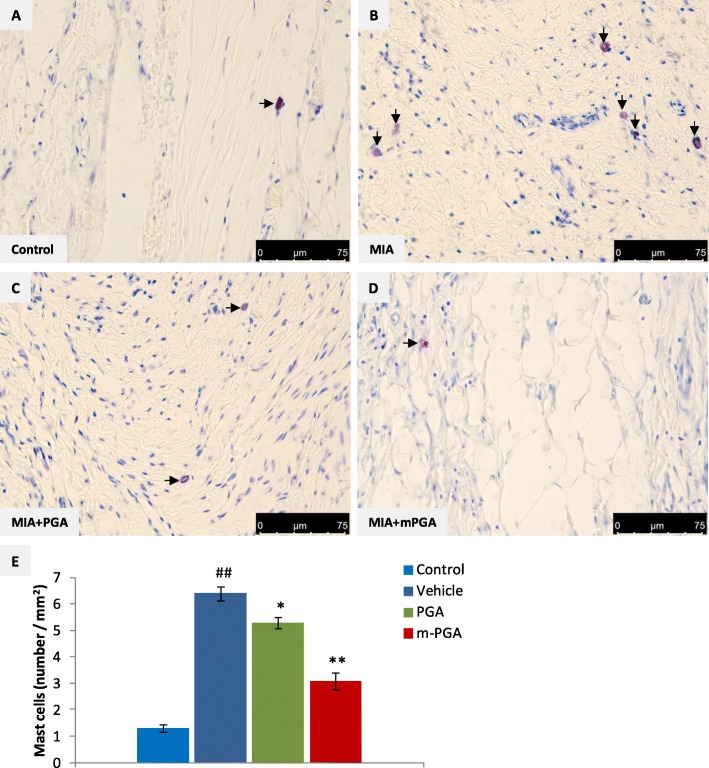
Effect of m-PGA on MIA-induced increase of mast cell density. Representative photomicrographs of toluidine blue-stained synovial sections. A quiescent mast cell densely packed with granules is visible (arrow) in the control group (**a**). Several degranulating mast cells were evident (arrows) in the synovium of MIA-injected rats (**b**). PGA (**c**) and m-PGA treatment (**d**) significantly decreased mast cell density (arrows) as confirmed by the statistical analysis (**e**). Results are expressed as means ± SEM (*N* = 10/group). ##*p* < 0.001 vs Control; *p < 0.05 vs vehicle; **p < 0.001 vs vehicle

Twenty-one days after MIA injection, knee sections showed a greater amount of both CD68-positive macrophages (Fig. 12a, b) and toluidine blue-positive mast cells compared to controls (Fig. 13a, b). The increase of the studied inflammatory cells was inhibited by PGA (Fig. 12c and Fig. 13c) and m-PGA (Fig. 12d and Fig. 13d). The analysis of mast cell density (i.e., the number of mast cells per square millimeter) confirmed the histologic observations and revealed a significant increase of joint mast cells in response to MIA injection (Fig. 13e). Conversely, mast cell number was significantly reduced in the joints of animals treated with PGA (*p* < 0.0154) and even more so with m-PGA (*p* < 0.001, Fig. 13e).

## Discussion

The present study showed m-PGA is safe, with LD50 ≥ 2000 mg/kg, i.e., 20 to 66 fold higher than the dose having beneficial therapeutic effects. In particular, the effects on CAR-induced inflammatory responses induced by a single oral administration of m-PGA were comparable to those of dexamethasone (particularly for the higher PGA dose). The finding is consistent with the results of previous studies which found that PGA endogenous congener, PEA (especially in micronized formulations) or the inhibitor of its degradative enzyme (i.e., the NAAA inhibitor ARN726) showed anti-inflammatory effects, which were comparable in efficacy to those exerted by dexamethasone [40–42]. The superior efficacy shown by micronized over non-micronized PGA in CAR-induced inflammatory responses was even more evident in OA-associated mechanical allodynia. This was shown by the significantly superior effect of m-PGA over PGA on MIA-induced decrease in pain withdrawal threshold and latency. In our view, this is a very interesting point since allodynia is a prominent feature of neuropathic pain, which represents a debilitating form of treatment-resistant, and possibly OA-related, chronic pain [43–45]. The superior effect of the tested micronized formulation is in agreement with previous studies which showed that reducing particle size of PGA parent molecule, PEA, highly and significantly increased both bioavailability and pain-relieving effect [25, 26]. Moreover, the observed effects on MIA-induced allodynia might suggest that possible mechanisms on peripheral and central sensitization are involved in m-PGA effect, similarly to those recently described for m-PEA in the experimentally inflamed temporomandibular joint [46]. It is interesting to note that oral supplementation with m-PGA in MIA-injected animals resulted in an anti-allodynic effect similar to that recently seen with intra-articular injection of PEA [47, 48]. The latter treatment option also benefited locomotor activity in MIA-injected rats [48], similarly to what was found here in orally administered animals. Of note, m-PGA resulted in a superior and earlier locomotor improvement compared to naïve non-micronized PGA. The MIA model is a well-established model of OA for both pain-related behaviors as well as histopathological changes [36, 49, 50]. Besides reducing allodynia and improving locomotor function in MIA-injected rats, PGA and even more so m-PGA also limited histological and radiographic damage and protected against MIA-induced loss of proteoglycans and collagen. The findings are consistent with those of a previous study reporting that the ALIAmide adelmidrol (a further PGA congener) limited MIA-induced cartilage degeneration and suchondral bone changes [51].

Finally, the present study showed that PGA significantly counteracted MIA-induced increase in joint inflammatory cells and plasma levels of IL-1β, TNF-α, metalloproteases, and NGF. Interestingly, hyperactive synovial cells (e.g., mast cells and macrophages) and chondrocytes are considered to the main sources of these pro-inflammatory and degradative bioactive mediators [7, 52–54]. The effect of PGA observed in the present study is thus consistent with the ALIA mechanism (i.e., down-modulation of cell hyperactivity) shown to be the main mechanism of action of the ALIAmide parent molecule PEA [11, 55, 56] and its congeners, i.e., adelmidrol [57] and PGA itself [18]. Once again, m-PGA showed superior efficacy over PGA in limiting the increase in the serum levels of IL-1β, TNF-α, metalloproteases, and NGF. Given the role played by the latter neurokine in OA pain [58, 59], the superior effect of m-PGA might explain its greater anti-allodynic effect over the non-micronized formulation.

There are multiple potential limitations to this study. The first pertains to the MIA model that is generally regarded as not accurately representing early pathophysiological alterations of human OA, while being considered one of the preferred models when studying OA pain [60]. Actually, representative histopathological findings of OA were recently shown in MIA-injected knees [36, 61] thus overcoming—at least in part—the potential limitation. The second is the lack of a glucosamine-treated group for reference. To the best of our knowledge, only one study has been published on glucosamine in the MIA-induced OA model and oral daily dose of 100 mg/kg for a 2-month duration was needed to show beneficial effects [23]. Different preclinical OA models were also used to evaluate glucosamine efficacy, the active daily dose ranging from 20 mg/kg to up to 1000 mg/kg (reviewed by Henrotin and coll [24].). In the present study, the dose used—and proved to be effective—was lower, i.e., 30 mg/kg PGA either micronized or not, corresponding to a glucosamine equimolar dose of 15 mg/kg. Moreover, the treatment frequency/duration was shorter (three times per week for 3 weeks) compared to the aforementioned OA preclinical studies on therapeutic potential of glucosamine. Although a glucosamine-treated group was actually lacking from our experiments, from the above one could conceivably reject the hypothesis that the observed effects were solely dependent on glucosamine released from PGA.

## Conclusion

Overall, the results of the present study showed that PGA had a global benefit, limiting joint inflammation, pain, and tissue damage, confirming preliminary findings by Costa and colleagues [19]. Moreover, it proved that particle size reduction greatly enhances the activity of the lipophilic compound PGA on joint pain and locomotor disability. Given these results, m-PGA could be considered a valuable option to concurrently counteract the pathogenic triad in OA (i.e., chondrodegeneration, inflammation, and pain). Clinical studies in human and veterinary patients are warranted to further evaluate therapeutic potential for m-PGA in naturally occurring OA.

## Supplementary information

**Additional file 1:** **Figure S1.** Histological evaluation after oral toxicity study. H/E representative pictures of brain, heart, lung stomach and colon of vehicle (A-E) or m-PGA group (F-J). No important histological alterations compared to vehicle-treated group were observed.

**Additional file 2:** **Figure S2.** Histological evaluation after oral toxicity study. H/E representative pictures of bowel, liver, kidney, bladder, uterus and spinal cord of vehicle (A-F) or m-PGA group (G-L). No important histological alterations compared to vehicle-treated group were observed.

## Data Availability

The datasets generated and/or analyzed for the present study are available from the corresponding author on reasonable request.

## Abbreviations

ALIA: Autacoids local injury antagonism
CAR: Carrageenan
Dex: Dexamethasone
IL: Interleukin
MIA: Sodium monoiodoacetate
MMP: Metalloprotease
mPGA: Micronized *N*-palmitoyl-d-glucosamine
MPO: Myeloperoxidase
NAAA: *N*-Acylethanolamine acid amidase
NGF: Nerve growth factor
OA: Osteoarthritis
PEA: *N*-Palmitoyl ethanolamine
PGA: *N*-Palmitoyl-d-glucosamine
PWL: Pain withdrawal latency
PWT: Pain withdrawal threshold
TNF: Tumor necrosis factor
