# Supplementary figures and images for: Safety and efficacy of a new micronized formulation of the ALIAmide palmitoylglucosamine in preclinical models of inflammation and osteoarthritis pain
Source: Arthritis Res Ther. 2019 Nov 28;21:254. doi: 10.1186/s13075-019-2048-y (PMC6883534; doi:10.1186/s13075-019-2048-y)

Vehicle

m-PGA

Brain

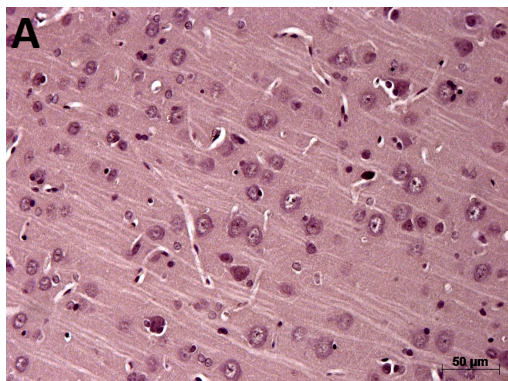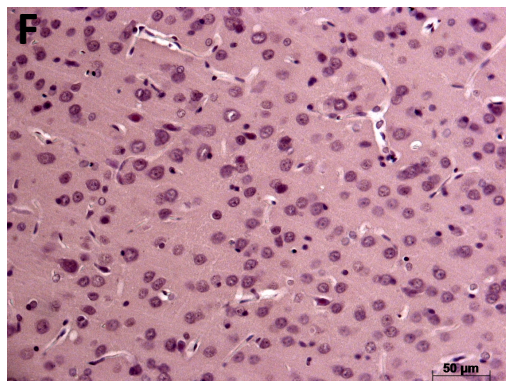

Heart

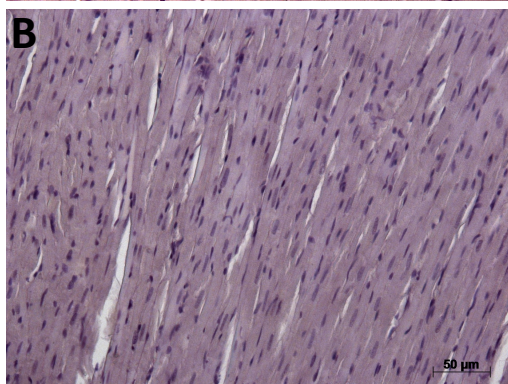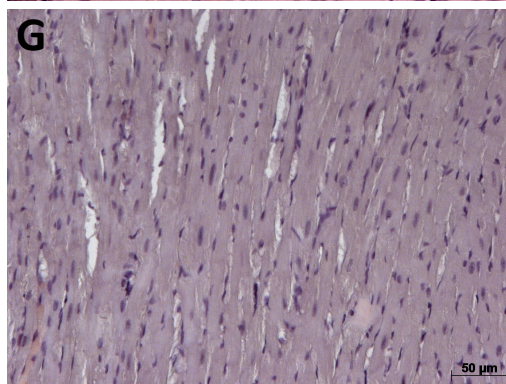

Lung

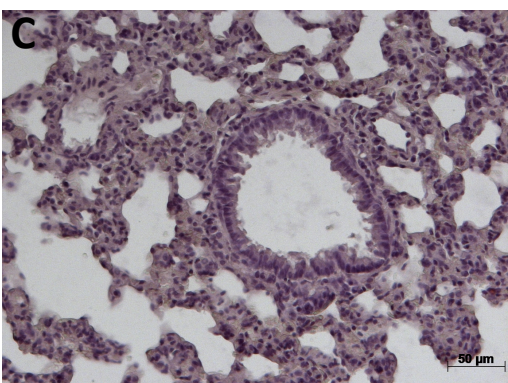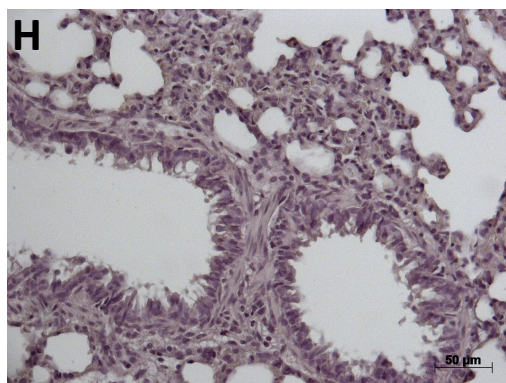

Stomach

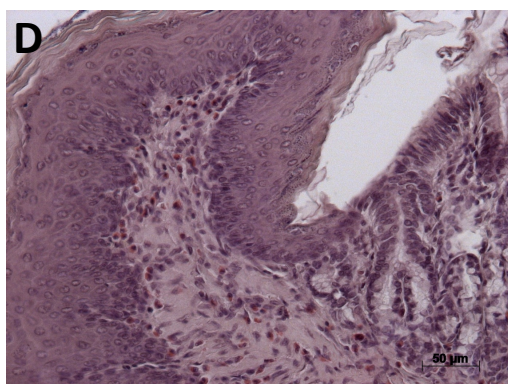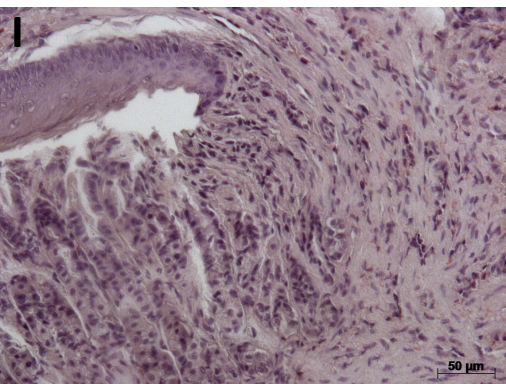

Colon

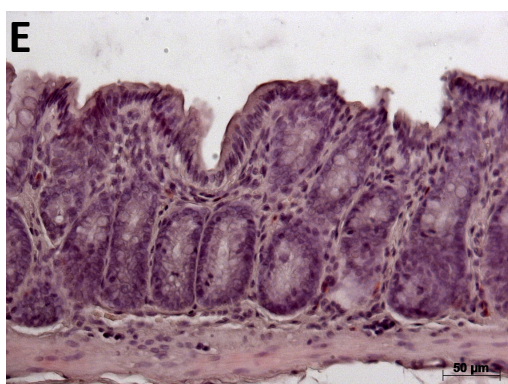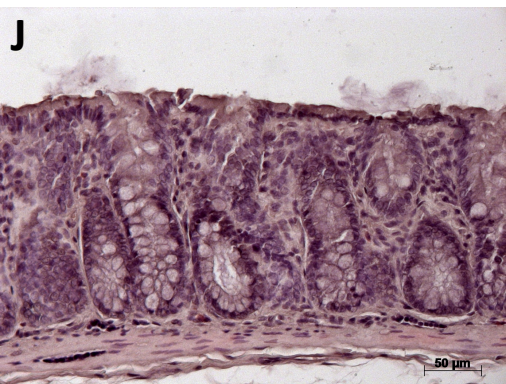

Supplementary Figure 1

Supplement: Supplementary file 1 — Additional file 1: Figure S1. Histological evaluation after oral toxicity study. H/E representative pictures of brain, heart, lung stomach and colon of vehicle (A-E) or m-PGA group (F-J). No important histological alterations compared to vehicle-treated group were observed. [file 13075_2019_2048_MOESM1_ESM.pdf]

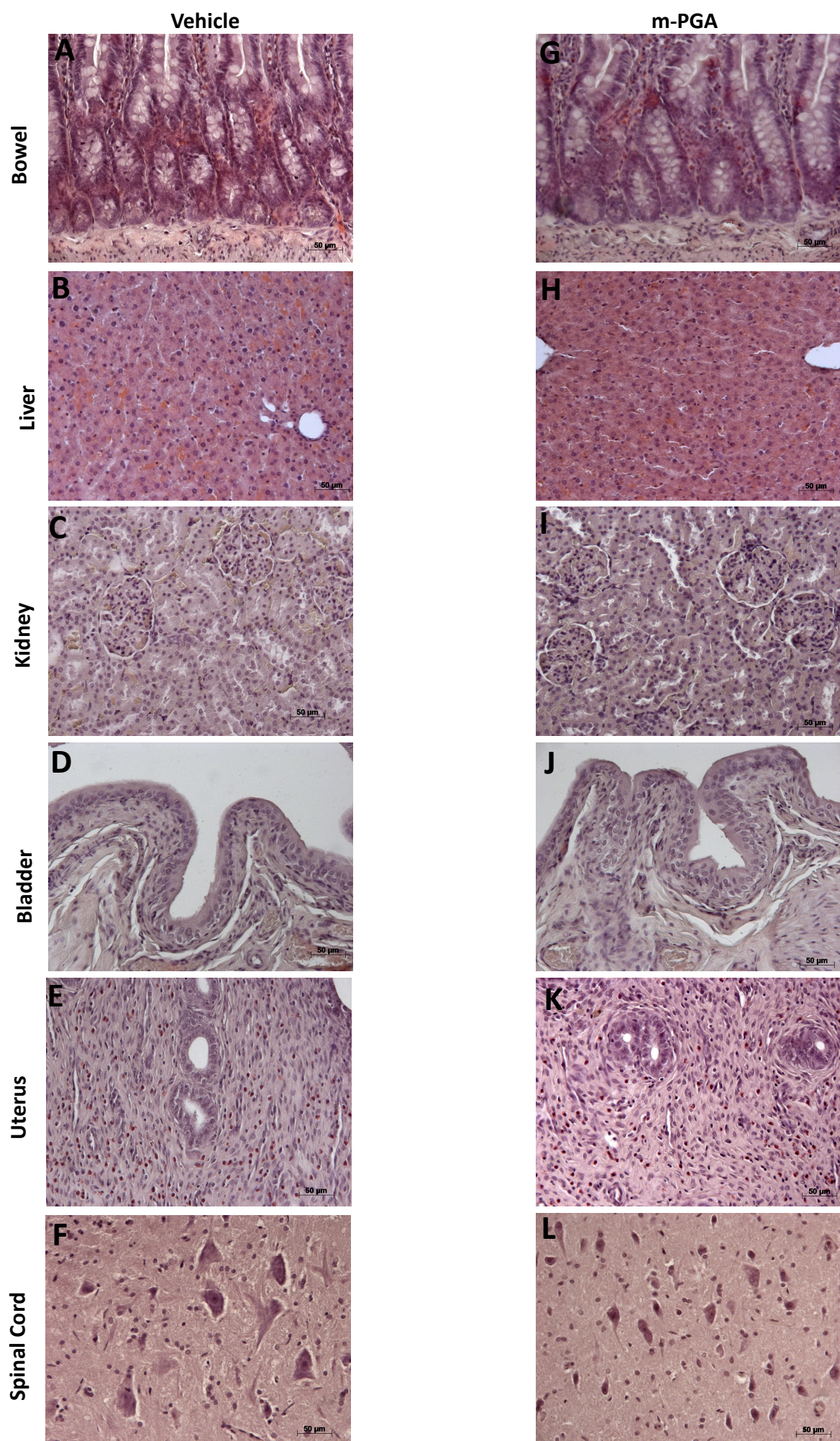

Supplementary Figure 2

Supplement: Supplementary file 2 — Additional file 2: Figure S2. Histological evaluation after oral toxicity study. H/E representative pictures of bowel, liver, kidney, bladder, uterus and spinal cord of vehicle (A-F) or m-PGA group (G-L). No important histological alterations compared to vehicle-treated group were observed. [file 13075_2019_2048_MOESM2_ESM.pdf]
